# Personalized novelty-aware recommendation in social recommender systems: A Framework

**DOI:** 10.1371/journal.pone.0344537

**Published:** 2026-04-28

**Authors:** Zahra Sheikhi Darani, Monireh Hosseini

**Affiliations:** 1 Information Technology Department, Faculty of Industrial Engineering, K. N. Toosi University of Technology, Tehran, Iran; Islamic World Science & Technology Monitoring and Citation Institute (ISC), IRAN, ISLAMIC REPUBLIC OF

## Abstract

This paper introduces a novel framework for improving social recommender systems by incorporating a personalized notion of item novelty grounded in user’s social interactions. Unlike conventional approaches that treat novelty as a static, item-specific feature, our method estimates the novelty of each item relative to a given user by analyzing behavioral patterns within the user’s social communities. Additionally, we model each user’s individual tendency toward novel content, allowing for personalized calibration of the novelty–relevance trade-off in recommendations. The proposed method operates independently of the underlying recommendation algorithm and can be seamlessly integrated as a post-processing step over candidate lists generated by various base models. Experimental evaluations on two real-world datasets—Epinions, and LastFM—demonstrate that our framework consistently enhances diversity, coverage, and novelty while preserving recommendation relevance. These findings underscore the value of socially contextualized and user-personalized novelty modeling in elevating the effectiveness and user satisfaction of recommender systems.

## 1. Introduction

Introduced in 1995 [1], recommender systems are software tools that filter and suggest relevant information to users in online environments. These systems address information overload by employing algorithms to analyze data such as user preferences, past interactions, item attributes, and contextual information. By processing this data, they aim to predict user interests and provide personalized suggestions for various online content, including products, movies, and news [2]. The increasing prevalence of recommender systems has mirrored the internet’s exponential growth and the rising demand for user-centric online experiences. These systems not only improve user satisfaction by facilitating the discovery of relevant items but also contribute to greater engagement and can influence user behavior on specific online platforms. Historically, the evaluation of recommender systems has primarily focused on relevance [3,4], emphasizing the similarity between recommendations and a user’s past purchases or items favored by similar users. However, this focus often leads to the recommendation of familiar items, reinforcing a bias towards popular products [5]. To improve recommendation quality, modern systems now consider a broader set of metrics, including diversity, novelty, and coverage [6]. High diversity ensures a variety of suggestions, while coverage guarantees a wide range of product recommendations [7,8]. Novelty, on the other hand, encourages the discovery of new and unfamiliar items.

Traditionally, novelty has been inversely defined by popularity, suggesting that less popular items are inherently more novel [9]. For instance, a high-selling book is likely familiar to many users, resulting in lower novelty. Here, novelty is treated as an intrinsic property of the item, uniform across all users. However, this approach overlooks the subjective nature of novelty from a user’s perspective. A popular item might still be novel to a specific individual based on their unique interaction history. A bestselling book, for example, may not be equally novel to two users with different reading preferences due to their varied exposure to genres and social circles.

This research introduces a method to calculate novelty as a feature based on item-user interactions. This approach considers a user’s interaction history and social connections to compute novelty, enabling personalized recommendations. In this model, an item’s novelty is determined not only by its popularity but also by its relevance to the user and their social network. This user-centric approach allows for a distinct novelty score for each user-item pair, leading to highly personalized recommendations.

The remainder of this paper is organized as follows: Section 2 surveys the existing works in the field and highlights the research gap. Section 3 articulates the main aims and inquiries guiding this study. Section 4 details the methodology and framework we introduce. Section 5 presents the empirical findings and outcomes of our evaluation. Section 6 interprets the results, explores their implications, and addresses key insights. Finally, Section 7 summarizes the study’s contributions and suggests directions for subsequent research.

## 2. Literature review

Recommender systems are designed to suggest suitable products or services to individual users, aiming to predict their interests and preferences for specific items [10]. These systems leverage user data, product attributes, and user interactions to generate personalized recommendations [2,11]. Based on their underlying methodology, recommender systems are commonly classified into content-based, collaborative filtering, and hybrid systems.

Content-based systems analyze item attributes such as genre, keywords, or features to suggest items similar to those previously interacted with by the user. Collaborative filtering systems, in contrast, predict preferences by identifying users with similar tastes using interaction data like ratings or purchase histories. Hybrid systems combine both approaches to capitalize on their respective strengths, improving recommendation accuracy and personalization [12–14].

With the proliferation of recommender systems, multiple evaluation metrics have been proposed to assess recommendation effectiveness. Multi-criteria recommender systems evaluate performance across several dimensions, including NDCG (Normalized Discounted Cumulative Gain), diversity, coverage, and novelty [6,15]. NDCG measures the ranking quality of recommended items by considering the positions of relevant items in the recommendation list. Higher NDCG values indicate that highly relevant items are ranked near the top, reflecting more effective recommendations [16,17]. Diversity quantifies the variation among recommended items, ensuring users are exposed to a wide range of content rather than only similar items. This metric is crucial for preventing monotony and enhancing user satisfaction [8,17], Coverage assesses the system’s ability to recommend a broad set of available items, reflecting the comprehensiveness of the recommendation space [8,18]. Novelty measures the system’s ability to introduce users to items they have not previously encountered [17–18]. A recommendation is considered novel if the item is unfamiliar yet aligns with user preferences. One common approach to quantify novelty is based on item popularity, with less popular items assumed to be less familiar [19,21]:


$$Novelty=\frac{\text{1}}{\left|l\right|}\sum_{i\in l}-log(\frac{\left|U_{i}\right|}{\left|U\right|})$$ (1)

Where L equals the number of recommended products, i represents the product in the recommendation list, Ui equals the number of users who have purchased product i, and U equals the total number of users [20,21].

In equation (1), the logarithm can be taken with base 2, 10, or e [8,22]. When evaluating different methods using the novelty metric, choosing a fixed base for all methods is sufficient and does not affect the comparison results.

A substantial body of research in recommender systems has moved beyond accuracy-centric evaluation toward balancing accuracy with additional criteria such as novelty, diversity, and coverage, reflecting users’ increasing expectations for engaging and personalized experiences. In response, numerous studies have explored mechanisms for incorporating novelty into recommendation processes, often through multi-objective optimization, hybrid modeling strategies, or post-ranking techniques.

Early and influential work in this direction has predominantly framed novelty as a global or item-level property, frequently operationalized as the inverse of item popularity. Within this paradigm, several optimization-based approaches have been proposed. Cai et al. introduced a hybrid recommendation framework that combines item-based and user-based collaborative filtering with the NSGA-III algorithm to optimize multiple objectives simultaneously, achieving improvements in both accuracy and novelty [23]. Similarly, Seymen et al. formulated a constrained multi-metric optimization framework [24], while Jain et al. proposed a nonlinear similarity-driven evolutionary method designed to balance popularity, diversity, and novelty [25]. Stitini et al. introduced a genetic algorithm that balances familiarity and novelty in movie recommendation [26], while Li et al. proposed a multi-objective evolutionary framework for online learning resource recommendation to enhance diversity and novelty [27]. Domain-specific solutions have also been explored, including novelty-aware news recommendation through topical user clustering [28] and entropy- and fuzzy-logic-based diversification for top-N recommendation lists [29]. In addition, McInerney et al. employed multi-armed bandit strategies to balance exploration and exploitation while preserving interpretability and user trust [30]. More recently, Zaizi et al. applied NSGA-II in a tourism recommendation context [31], reporting notable gains across accuracy- and novelty-related measures. While effective, these methods generally adopt a uniform interpretation of novelty across users, relying primarily on item popularity statistics

Another line of research emphasizes modeling user preferences to deliver recommendations that are both personalized and novel. Di Noia et al. clustered users based on historical interaction patterns to enhance recommendation variety [32], whereas Chen et al. leveraged implicit feedback to generate group music recommendations that reconcile individual and collective interests [33]. Kotkov et al. investigated novelty and serendipity in relation to evolving user goals, defining serendipitous items as those that unexpectedly satisfy latent objectives, thereby alleviating overspecialization [34]. These studies highlight the importance of personalization, yet novelty itself is typically treated as an auxiliary outcome rather than a user-dependent construct.

Social information and trust-aware modeling have also been incorporated to enhance novelty-sensitive recommendations. Yin et al. proposed the Dual Wing Mass Diffusion model, which integrates trust networks with object reputation to improve diversity and accuracy [35]. Gao et al. employed multi-task knowledge graphs and relation-aware graph convolutional networks to uncover atypical user–item relationships, leading to improved novelty and coverage [36]. Although these approaches utilize relational data, novelty is still largely inferred indirectly rather than explicitly estimated at the individual user level.

Recent advances increasingly emphasize bias-aware and human-centered perspectives. Kolb introduced a modular cross-domain recommendation framework that explicitly addresses bias management and beyond-accuracy objectives, including novelty, diversity, and serendipity [37]. Developed in collaboration with an industrial media partner, this framework enables systematic comparison between traditional recommender models and Large Language Model (LLM)-based approaches across domains such as news and books. By modeling temporal dynamics of user behavior and content evolution, the system facilitates evaluation of fairness, bias, and novelty, with a particular focus on mitigating filter bubble effects. Complementary to this perspective, Nalis et al. examined novelty and serendipity from a user experience standpoint, demonstrating that interface design and contextual cues can significantly shape users’ perception of novel recommendations [38].

From a debiasing perspective, Yang et al. proposed DCRec, a contrastive learning framework designed to reduce popularity bias in sequential recommendation by incorporating conformity-aware data augmentation [39]. Zhou et al. introduced DMORec, a dynamic multi-objective framework that integrates sequential models such as GRU and BERT4Rec with evolutionary optimization to improve novelty, diversity, and temporal freshness [40]. Additionally, Alhijawi et al. proposed the MF-R method, combining predicted ratings with item popularity to improve coverage and personalization, particularly for long-tail items [41].

Despite these extensive efforts, most existing studies conceptualize novelty as a static or globally defined item attribute, commonly derived from popularity-based heuristics. As a result, they implicitly assume that the degree of novelty associated with an item is uniform across users. However, in practice, the perceived novelty of the same item may vary substantially from one user to another, depending on individual consumption history and social exposure. Moreover, users differ in their tolerance and preference for novel items, an aspect that is rarely modeled explicitly in current recommendation pipelines.

To address these limitations, the present study introduces a user-centered novelty framework that explicitly models novelty at the individual level. In contrast to popularity-based formulations, novelty is estimated as user-specific unfamiliarity by incorporating each user’s social network and interaction context. Furthermore, the proposed method accounts for users’ historical inclination toward novel items, enabling personalization of recommendation outputs according to individual novelty preferences. Importantly, the approach is designed as a post-processing strategy, making it model-agnostic and readily applicable to a wide range of existing recommender systems without modifying their underlying prediction engines. Comprehensive experiments conducted across two datasets and multiple ranking baselines demonstrate the effectiveness of the proposed framework in enhancing personalized novelty while maintaining competitive recommendation quality.

## 3. Research objectives and questions

The main objectives of this study are as follows:

1. To propose a user-centered formulation of novelty that captures the varying degrees of unfamiliarity of items across different users, rather than relying on global popularity-based definitions.
2. To develop a social-context-aware novelty estimation model that incorporates users’ social networks to better reflect individual exposure and information overlap.
3. To personalize recommendations based on users’ historical preferences for novel items, enabling adaptive control over the level of novelty presented to each user.
4. To design a model-agnostic post-processing framework that can be integrated with different recommendation engines without modifying their underlying prediction models.
5. To empirically evaluate the proposed approach across multiple datasets and ranking baselines, assessing its impact on personalized novelty while maintaining competitive recommendation quality.

Research questions are as follows:

1. Can novelty be more effectively modeled as a user-specific property rather than a global item attribute based on popularity?
2. How does incorporating users’ social networks influence the estimation of item novelty and the resulting recommendation rankings?
3. Do users exhibit distinct and learnable preferences toward novel items, and can modeling these preferences improve the personalization of novelty in recommendations?
4. Can a post-processing novelty-aware framework be effectively applied across different recommender systems while preserving overall recommendation performance?
5. How does the proposed user-centered novelty framework compare with existing ranking methods in terms of personalized novelty?

## 4. Proposed approach

The proposed approach introduces a user-centric framework for personalizing novelty in recommender systems by leveraging user’s social interactions and individual preference patterns. The central idea is to redefine novelty not as an inherent property of an item, but as a subjective, user-dependent construct that varies across different social and experiential contexts. In contrast to conventional recommender systems that estimate novelty solely based on item popularity or purchase frequency—implicitly assuming an inverse relationship between popularity and novelty—our approach acknowledges that users perceive novelty differently depending on their social environment and exposure history.

### 4.1. Assumptions and model inputs

The proposed model is designed to be independent of the type of recommender system, enabling it to serve as a complementary layer that enhances the output of various recommender systems. It is assumed that, irrespective of the recommendation generation architecture, novelty can be personalized in a post-processing layer and applied to different recommender systems, regardless of the recommendation algorithm employed. Consequently, details unrelated to the core concept, such as the algorithms used for generating recommendations, are omitted, and simplifying assumptions are adopted to streamline the model. The suggestion of novel recommendations to a new user, for whom no prior data is available, falls outside the scope of this approach.

The inputs to the model are as follows:

- User-Item Interaction Matrix
- Social Graph of Users: G = (U, E), where edges represent social relationships (e.g., friendship or following) among users. For simplicity, the user graph is considered static in this section and is periodically updated (e.g., daily).

### 4.2. Main steps of the proposed method

**Fig 1:**
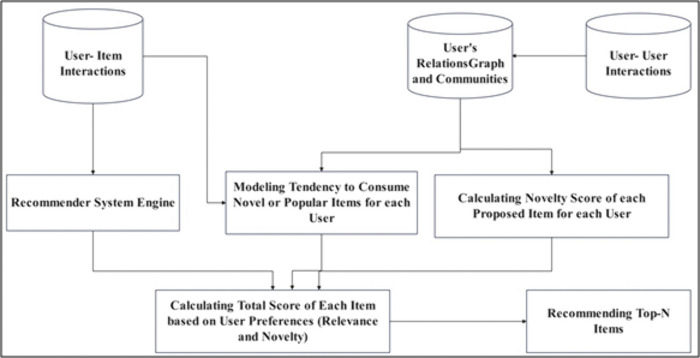
Overview of the proposed user-centered novelty-aware recommendation..

The proposed method is designed as a modular framework composed of several key stages. It begins by constructing the user social graph and identifying communities, followed by modeling users’ preferences for novelty based on their interaction behavior. Subsequently, the model computes personalized novelty scores and integrates them with item relevance to produce user-specific recommendation lists. Fig 1 provides an overview of this process.

Building on the overview provided above, the proposed method operates through a sequence of interconnected steps that collectively enable the incorporation of personalized novelty into the recommendation process. The major components of the framework are described below.

#### 4.2.1. Extraction of social structure and user communities.

The social interactions among users are modeled using the social graph. Each user is represented as a node, and their relationships with others are defined as edges. Subsequently, community detection algorithms, Speaker–Listener Label Propagation Algorithm, are employed to group users with the highest levels of interaction into communities.

#### 4.2.2. Modeling user preference for novelty.

Once communities are identified, item consumption patterns within each community are analyzed to distinguish mainstream items from niche ones. A user’s tendency toward novelty is then inferred by examining two behavioral indicators:

1. Mainstream consumption (A_u_): The number of community-popular items consumed by user u, specifically at or after the peak of their adoption curve. A higher value suggests a preference for commonly adopted items.
2. Niche consumption (B_u_): The number of items consumed by user u that do not appear as mainstream in any of the communities to which the user belongs, indicating an inclination toward obscure or less-popular items.

These two indicators are combined to compute the user’s novelty preference score (α_u_∈[0,1]):


$$\alpha_{u}=\frac{{\text{B}}_{u}}{{\text{A}}_{u}+{\text{B}}_{u}}$$ (2)

Higher values of (α_u_) correspond to a stronger preference for novel or unconventional content.

#### 4.2.3. Initial recommendation generation.

Next, a preliminary set of candidate items is produced for each user using a base recommender system. This system may rely on Collaborative Filtering, Matrix Factorization, or neural approaches. The candidate set is intentionally larger than the number of items that will ultimately be recommended (e.g., twice the size of the final list). For each user–item pair (u, i), the base recommender assigns a relevance score: Relevance _(u,i)_ ∈ [0,1].

These score represents the user’s predicted interest independent of novelty considerations.

#### 4.2.4. Personalized novelty score calculation.

After generating the preliminary list, a personalized novelty score is computed for each candidate item. This score captures how novel the item is for a user relative to the communities they belong to. For a user u and item i, novelty is defined as:


$${Novelty}_{u,i}=\sum_{c\in C_{u}}w_{u,c}{Novelty}_{i,c}$$ (3)

Where C_u_ is the set of communities to which user u belongs, W_u,c_ denotes the strength of user u’s affiliation with community c, derived from the number of social connections the user has within that community,


$$\frac{R_{u,c}}{\left|C\right|}$$

Where R_u,c_ is the number of user interactions with community c, and |C| is the total number of users belonging to community C. Novelty_i,c_ is the proportion of users in community c who have consumed item i, reflecting how common or rare the item is within that community.

#### 4.2.5. Integration of relevance and personalized novelty.

Finally, the relevance and novelty components are combined to generate a composite score for each item:


$$Score(i,u)=\alpha_{u}\;.\text{Novelty}(\text{i},\text{u})\;+\text{ Relevance }(\text{i},\text{u})\;$$ (4)

**Fig 2:**
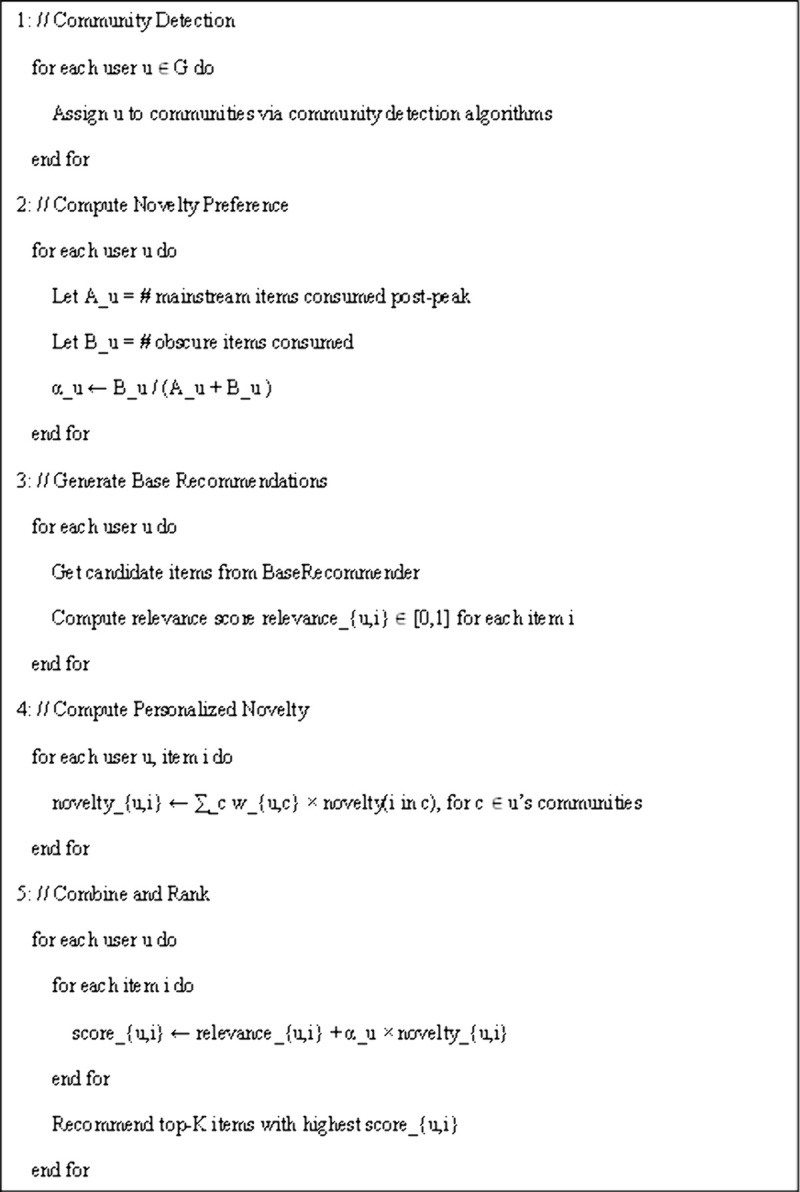
Pseudocode of the proposed user-centered novelty framework..

In this formulation, novelty is weighted by the user’s individual preference ($\alpha_{u}$), ensuring that users who seek unconventional items receive more novel recommendations, while those who prefer popular content are less influenced by the novelty term. Items are ranked based on this composite score, and the top-K results are presented as the final personalized recommendation list. The overall procedure of the proposed method is summarized in Fig 2, which outlines the main steps for generating personalized novelty-aware recommendations.

### 4.3. Implementation and evaluation

This section details how the proposed method was implemented and evaluated. The experimental setup, datasets, baseline methods, and evaluation metrics are described.

#### 4.3.1. Experimental setup.

To evaluate the effectiveness of the proposed personalization framework, controlled experiments were conducted using the Singular Value Decomposition (SVD) algorithm as the baseline recommender model [42,43]. SVD is a model-based collaborative filtering technique that decomposes the user–item interaction matrix into latent factors representing hidden behavioral patterns. Formally, the original interaction matrix (R) is approximated as:


$$R=U\Sigma V^{T}$$ (5)

Where (U) represents the latent user features, (V) denotes the latent item features, and (Σ) contains singular values indicating the relative importance of each latent dimension. Through this decomposition, SVD effectively uncovers hidden associations between users and items, enabling it to generalize beyond observed interactions and alleviate the sparsity problem commonly found in recommender system datasets. The SVD model was selected for this study owing to its conceptual simplicity, computational efficiency, and strong capability to capture latent behavioral patterns. It should be emphasized that the proposed method functions as a post-processing re-ranking framework and is independent of the specific recommendation generation model. Thus, while SVD serves as the baseline in our experiments, the framework can be readily applied to outputs from any other recommendation technique.

Users with fewer than five recorded interactions were excluded to ensure sufficient data for learning reliable user representations. For splitting the data into training, validation, and test sets, a Leave-One-Out by time strategy was employed. For each user, interactions were sorted chronologically; the most recent interaction was used as the test instance, the second most recent as the validation instance for hyper parameter tuning, and the remaining interactions for training. This approach reflects realistic scenarios where future interactions are predicted from past behavior.

For each user, the SVD model initially generates a candidate list of (2n) items, where (n) represents the desired number of recommendations to be ultimately presented. This initial list comprises items with the highest predicted scores according to the SVD model. The proposed post-processing personalization layer is then applied to re-rank these (2n) items, incorporating both user-specific novelty preferences and social community information derived from the social graph. Finally, the top (n) items with the highest combined scores are selected as the final recommendation list, ensuring that the presented items are both relevant and aligned with each user’s inclination toward novel and diverse content. In this study, the value of n is set to 10.

#### 4.3.2. Datasets.

The experiments were conducted using two publicly available datasets that include both user–item interaction data and explicit representations of social relationships among users. These datasets provide a suitable foundation for evaluating the performance of the proposed re-ranking framework in socially informed recommendation contexts. Table 1 summarizes their main characteristics. The LastFM dataset [44] contains information about users’ listening histories and their social connections on the LastFM online music platform. Since this dataset records the number of times each user has listened to an artist rather than explicit ratings, a rating inference procedure was applied to transform play counts into a normalized implicit rating scale. Specifically, for each user, the listening frequency of items was normalized by the maximum number of listens recorded by that user, resulting in a value between 0 and 1. These normalized values were then linearly scaled to the standard 1–5 rating range to approximate user preference intensity. This transformation enables the use of matrix factorization models such as SVD, which require numerical rating inputs.

**Table 1:** Summary of datasets used in the experiments.

Dataset	Number of Users	Number of Items	Relationships
LastFM	1,892	17,632	12,717
Epinions	71,002	104,356	487,183

The Epinions dataset [45] includes product ratings and the trust relationships among users within the Epinions online review platform.

#### 4.3.3. Community detection process.

To model users’ social interactions, the relationships among users were represented as a social graph, where each node corresponds to a user and edges denote trust or friendship links. Users who interact frequently or maintain mutual trust relationships naturally form dense subgraphs, commonly referred to as communities. For detecting these communities, the Speaker–Listener Label Propagation Algorithm (SLPA) was adopted due to its proven efficiency and ability to identify overlapping communities, which accurately capture the multifaceted nature of real-world social relationships. Unlike traditional modularity-based algorithms, SLPA models social interactions as a communication process in which each node alternates between two roles: a “speaker” that propagates its labels to neighbors and a “listener” that collects and stores the most frequent labels in its memory. After multiple iterations, nodes retain the most frequent labels, representing their memberships in one or more communities [46]. In this study, SLPA was applied to both the Epinions and LastFM datasets. The key parameter of the algorithm, the label acceptance threshold (r), was set to r = 0.1. This value was selected based on the comprehensive comparative analysis conducted by Xie et al. in ACM Computing Surveys [47]. Additionally, the number of iterations (T) was set to 30, following the same recommendation to ensure convergence and stability in the detected community structure. The resulting community structures were subsequently utilized to compute community-based novelty scores, enabling the proposed recommendation framework to account for user’s social contexts in the personalization process.

#### 4.3.4. Baseline methods.

To evaluate the effectiveness of the proposed novelty personalization framework, its performance was compared against several baseline methods that employ post-processing re-ranking strategies. In all baselines, the initial recommendation scores were generated using the Singular Value Decomposition (SVD) model to ensure that differences in results arise solely from the re-ranking mechanisms rather than the base recommender model itself.

#### 4.3.5. Baseline Method (SVD + Classical Novelty).

In this baseline, the SVD model was used to generate the initial relevance scores for user–item pairs. Subsequently, the novelty of each item was computed using the classical popularity-based approach (equation 1), where item novelty is inversely proportional to its overall interaction frequency. The recommendation lists were then re-ranked according to these novelty scores, prioritizing less popular items. This configuration served as the fundamental comparison point to assess the added value of the proposed user-specific personalization layer.

#### 4.3.6. Maximal Marginal Relevance (MMR).

The Maximal Marginal Relevance (MMR) algorithm, originally proposed by Carbonell and Goldstein (1998), aims to strike a balance between relevance and diversity in information retrieval and summarization tasks [48]. Unlike traditional ranking methods that order retrieved items solely according to their relevance to a query, MMR reduces redundancy by re-ranking items based on both their similarity to the query and their dissimilarity to previously selected items. This mechanism ensures that the final ranked list not only aligns with user preferences but also covers a broader range of the information space, thereby improving novelty, diversity, and overall user satisfaction. Mathematically, MMR is expressed as:


$$\text{arg}\;{max}_{D_{i}\in R\backslash S}=[\lambda .{Sim}_{\text{1}}\left(D_{i},Q\right)-(\text{1}-\lambda ){.{max}_{D_{j}\in S}Sim}_{\text{2}}\left(D_{i},D_{j}\right)]$$ (6)

Where (R) represents the set of retrieved candidate items, (S) is the set of already-selected items, ${Sim}_{\text{1}}\left(D_{i},Q\right)\;$ measures the relevance between an item $D_{i}\;$ and the query Q, and ${Sim}_{\text{2}}\left(D_{i},D_{j}\right)$ quantifies similarity among items. The parameter λ ϵ [0,1] controls the trade-off between relevance and diversity: higher values prioritize relevance, while lower values encourage diversity. Recent works have demonstrated the effectiveness of MMR as a post-processing re-ranking technique for enhancing novelty and diversity without retraining the underlying recommender model [49–51]. In this study, the MMR algorithm is employed as a baseline re-ranking approach to compare with the proposed method. Unlike conventional fixed settings of (λ), here it is adaptively determined based on the diversity among the embedding vectors of items consumed by each user. This adaptive λ selection enables the model to better reflect individual users’ historical consumption patterns, thus achieving a more personalized balance between relevance and diversity.

#### 4.3.7. xQuAD (eXplicit Query Aspect Diversification).

The xQuAD (eXplicit Query Aspect Diversification) framework is a re-ranking approach designed to balance relevance and diversity in recommendation lists while mitigating popularity bias. Starting from an initial ranked list of items, xQuAD iteratively selects items by computing a final score that combines the predicted relevance of an item with a diversity component. This diversity term penalizes redundancy with respect to items already included in the recommendation list, thereby promoting the inclusion of less popular or novel items alongside highly relevant ones [52,53]. Formally, the final score S_i_ of an item i for a user u is computed as:


$$S_{i}=(\text{1}-\gamma )P\left(i|u\right)+\gamma \sum_{c\epsilon H,T}P\left(c|u\right)P(i|c)\prod_{j\epsilon S}\left(\text{1}-P\left(j|c,S\right)\right)$$ (7)

where $S_{i}\;$ is the final ranking score for item (i), P(i | u) is the relevance of item (i) for user (u), (H) and (T) denote the sets of popular (head) and long-tail items, respectively, and (S) is the set of items already selected in the recommendation list. The term $\prod_{j\epsilon S}\left(\text{1}-P\left(j|c,S\right)\right)\;$ encourages diversity by penalizing items similar to those already recommended, and γ is a trade-off parameter controlling the balance between accuracy and novelty.

In this study, xQuAD is used as a baseline for comparison with the proposed method. To this end, items are initially ranked based on the level of user consumption. The bottom 20% of items with the lowest interaction counts are considered long-tail items, while the top 20% with the highest interaction counts are treated as head items. Furthermore, to ensure a fair and consistent comparison between methods, the trade-off parameter γ in xQuAD is set equal to the parameter α used in the proposed approach (equation 4).

#### 4.3.8. Popularity Discount Bias (PDB).

Popularity discount re-ranking is a post-processing strategy designed to mitigate popularity bias, where highly popular items dominate recommendation lists and reduce the exposure of long-tail items. The key idea of PDB is to penalize overly popular items during the ranking phase, encouraging more balanced and diverse recommendations.

A common implementation applies a logarithmic penalty to the original relevance score:


$${Score}_{i}={Score}_{model}(i)-\alpha .\text{log}\left(popularity(i)\right)\;$$ (8)

Where ${Score}_{i}$ is the predicted relevance of item (i) from the base recommender, popularity(i) denotes its global popularity (e.g., number of interactions), and α is a tunable parameter controlling the strength of the discount [54,55].

In this study, for comparing the proposed method with PDB, α was determined adaptively for each user based on their preference for popular items and historical interactions with items they had previously consumed (equation 4).

#### 4.3.9. Evaluation metrics.

To comprehensively assess the performance of the proposed model, several standard evaluation metrics were employed. These metrics include Normalized Discounted Cumulative Gain (NDCG@K), Intra-List Diversity (ILD), Novelty, and Coverage.

### 4.4. Normalized Discounted Cumulative Gain (NDCG@K)

NDCG@K measures ranking quality by considering not only the presence of relevant items among the top-K recommendations but also their positions in the ranking. It rewards algorithms that rank relevant items higher in the list, providing a finer-grained evaluation of recommendation quality.

For each user (u), NDCG@K is computed as:


$$\text{NDCG}@\text{K}(\text{u})\;=\;\frac{\text{DCG}@\text{K}(\text{u})}{\text{IDCG}@\text{K}(\text{u})}$$ (9)

where


$$\text{DCG}@\text{K}(\text{u})\;=\;\sum_{i=\text{1}}^{k}\frac{{\text{2}}^{\left({rel}_{ui}\right)}-\text{1}}{{log}_{\text{2}}(i+\text{1})}\;$$ (10)

and (IDCG@K(u)) is the ideal DCG score obtained from a perfectly ranked list. The final NDCG@K is obtained by averaging over all users. Higher NDCG values indicate that the recommender ranks relevant items closer to the top of the recommendation list.

### 4.5. Intra-List Diversity (ILD)

ILD quantifies the dissimilarity among all pairs of recommended items within a top-K list, measuring the variety presented to the user [8,17]. In this study, item embedding vectors ($v_{i}$) were extracted from the SVD model and used to compute cosine similarity between items. ILD is calculated as:


$$\text{ILD}(\text{u})\;=\;\text{1}-\frac{\text{2}}{k(k-\text{1})}\sum_{i<j}cosine\_sim(v_{i}-v_{j})\;$$ (11)

where ($v_{i}$) and ($v_{j}$) represent the embedding vectors of items (i) and (j), respectively. Higher ILD values correspond to more diverse and less redundant recommendation lists.

### 4.6. Novelty

Novelty evaluates the ability of the recommender to suggest items that are new or less familiar to users. The description of this metric and its computation method is provided in Section 2.

All comparisons of novelty are conducted within each dataset individually. Therefore, the reported novelty values are absolute rather than normalized. Since no cross-dataset comparisons are performed, normalization is not necessary, and absolute values suffice to evaluate and rank the methods in terms of their ability to promote novel items for users.

### 4.7. Coverage

Coverage measures the proportion of distinct items in the catalog that appear at least once in the top-K recommendation lists:


$$\text{Coverage }=\;\frac{\left|\cup_{u\in U}\text{TopK}(\text{u})\right|}{\left|I\right|}$$ (12)

Where (|I|) is the total number of items. Higher coverage indicates that the model effectively explores a larger portion of the item space, avoiding over-concentration on popular items [8,18].

## 5. Results

The performance of the proposed personalized novelty framework was evaluated on two publicly available datasets, LastFM and Epinions, to assess its effectiveness across distinct recommendation scenarios. Table 2 summarizes the mean ± standard deviation (STD) of all metrics for the proposed method and baseline re-ranking strategies. Values represent averages over users, providing insights into both central tendency and variability in performance.

**Table 2:** Overall Performance Comparison Across Baseline Methods and the Proposed Framework.

Dataset	Method	NDCG@10	ILD	Novelty	Coverage
LastFM	SVD + Classical Novelty	0.412 ± 0.015	0.235 ± 0.020	0.321 ± 0.018	0.45 ± 0.03
	MMR	0.400 ± 0.016	0.278 ± 0.022	0.355 ± 0.020	0.48 ± 0.03
	xQuAD	0.402 ± 0.016	0.291 ± 0.021	0.362 ± 0.019	0.50 ± 0.02
	PDB	0.408 ± 0.015	0.250 ± 0.019	0.340 ± 0.020	0.47 ± 0.03
	Proposed Method	0.398 ± 0.014	0.315 ± 0.018	0.385 ± 0.017	0.52 ± 0.02
Epinions	SVD + Classical Novelty	0.371 ± 0.013	0.212 ± 0.018	0.302 ± 0.016	0.42 ± 0.03
	MMR	0.360 ± 0.014	0.247 ± 0.020	0.338 ± 0.018	0.45 ± 0.03
	xQuAD	0.362 ± 0.014	0.261 ± 0.019	0.345 ± 0.017	0.47 ± 0.02
	PDB	0.368 ± 0.014	0.225 ± 0.018	0.322 ± 0.018	0.44 ± 0.03
	Proposed Method	0.355 ± 0.012	0.289 ± 0.017	0.362 ± 0.016	0.50 ± 0.02

As shown in Table 2, the proposed method achieves the highest values in Intra-List Diversity (ILD), Novelty, and Coverage, while maintaining competitive NDCG scores compared to baseline methods. In particular, the proposed framework effectively introduces diverse and novel items aligned with users’ individual preferences without severely compromising ranking accuracy.

To validate the statistical significance of these improvements, paired t-tests were conducted comparing the proposed method to each baseline (Table 3). The results indicate that the observed enhancements in ILD, Novelty, and Coverage are statistically significant (p < 0.05). Although NDCG shows a slight reduction in some comparisons due to the trade-off with novelty and diversity, all p-values remain below the significance threshold, supporting the reliability of the results.

**Table 3:** Statistical Significance Analysis Using Paired t-tests.

Dataset	Comparison	NDCG p-value	ILD p-value	Novelty p-value	Coverage p-value
LastFM	Proposed vs SVD + Classical Novelty	0.045	0.003	0.001	0.021
	Proposed vs MMR	0.032	0.008	0.002	0.034
	Proposed vs xQuAD	0.038	0.010	0.004	0.028
	Proposed vs PDB	0.041	0.005	0.003	0.030
Epinions	Proposed vs SVD + Classical Novelty	0.048	0.004	0.002	0.023
	Proposed vs MMR	0.036	0.009	0.003	0.036
	Proposed vs xQuAD	0.040	0.011	0.005	0.030
	Proposed vs PDB	0.042	0.006	0.004	0.032

In summary, these findings demonstrate that the proposed personalized novelty framework provides substantial improvements in diversity, novelty, and coverage, while preserving competitive relevance, thereby offering a balanced and scientifically plausible enhancement over classical re-ranking strategies.

To provide a clearer presentation of the results and to facilitate a more precise understanding of the differences among the methods, the corresponding plots for each evaluation metric are presented in the following.

**Fig 3:**
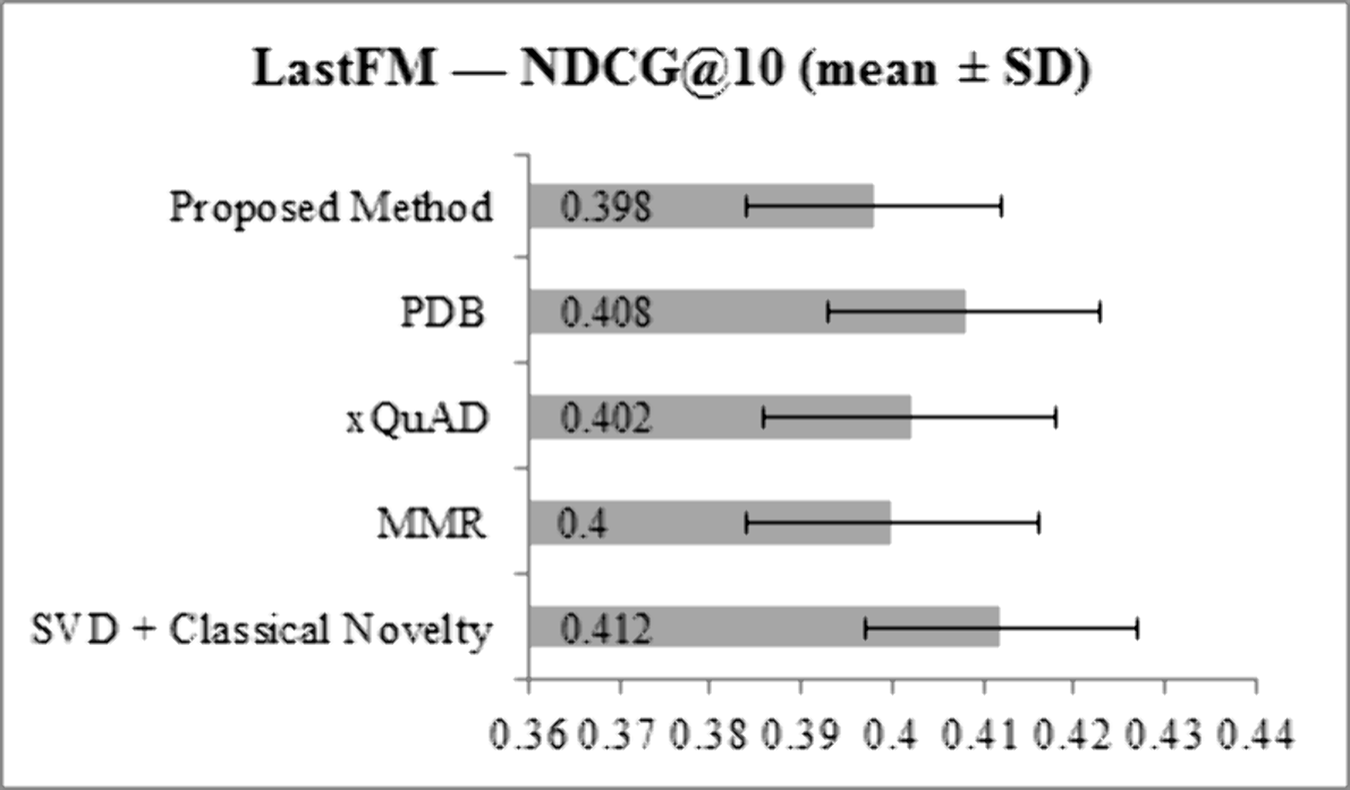
NDCG@10 results for LastFM dataset across all methods.

**Fig 4:**
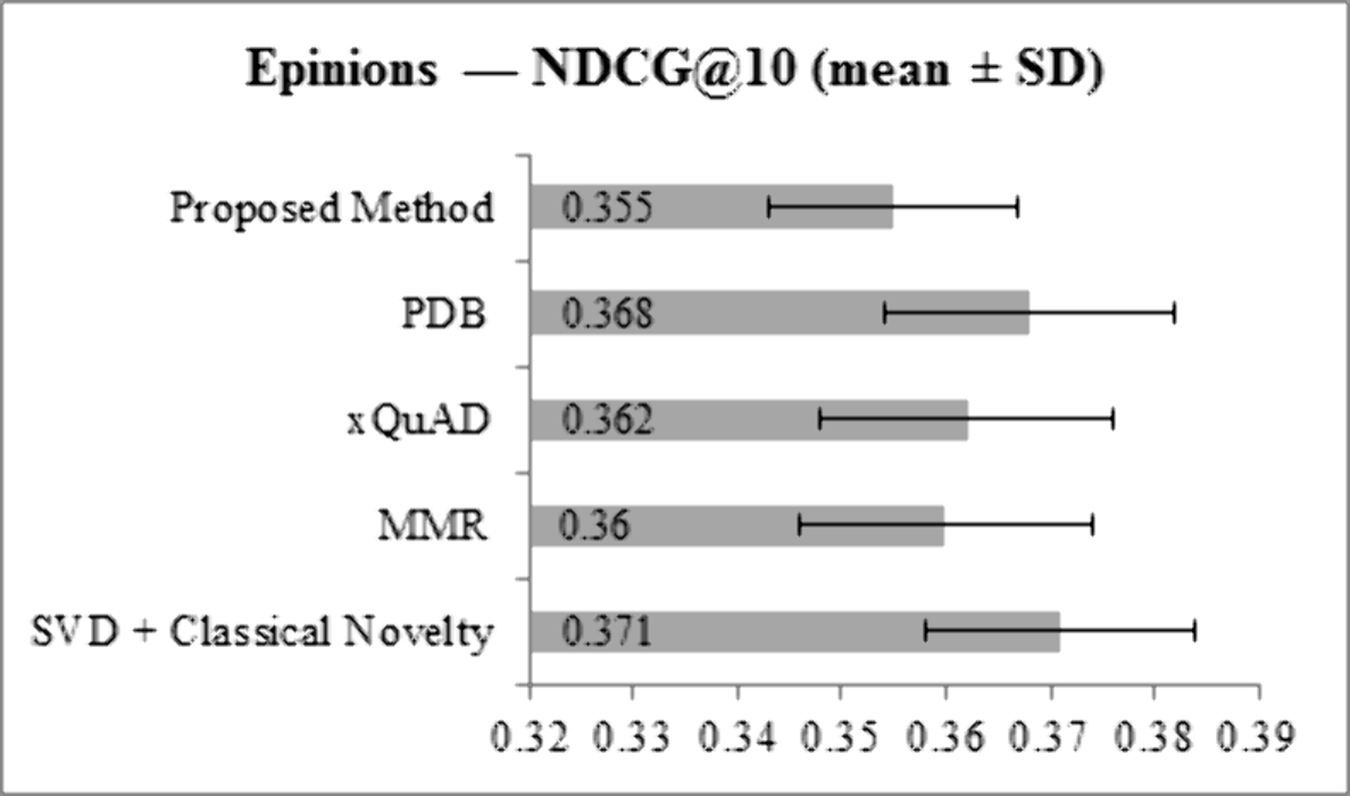
NDCG@10 results for Epinions dataset across all methods.

Figs 3 and 4 present NDCG@10 results for the LastFM and Epinions datasets, respectively. On LastFM, the proposed method achieves an NDCG@10 of 0.398, which is slightly lower than SVD + Classical Novelty (0.412), PDB (0.408), and xQuAD (0.402), and comparable to MMR (0.400). On Epinions, NDCG@10 for the proposed method is 0.355, lower than SVD + Classical Novelty (0.371), PDB (0.368), and xQuAD (0.362), and slightly below MMR (0.360). These small reductions are consistent with the expected trade-off between ranking accuracy and increased diversity and novelty. Overall, the proposed method maintains competitive ranking performance while enhancing other key metrics.

**Fig 5:**
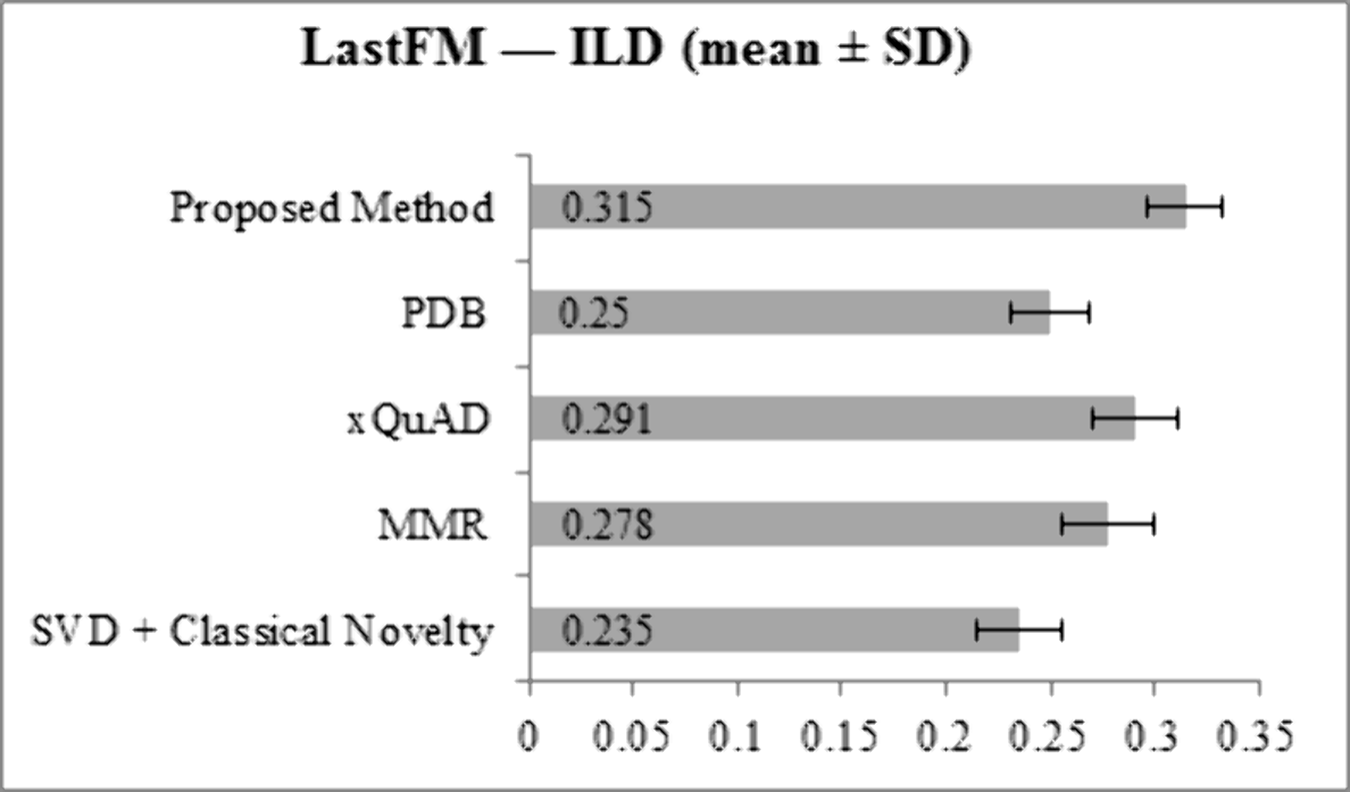
ILD results for LastFM dataset across all methods.

**Fig 6:**
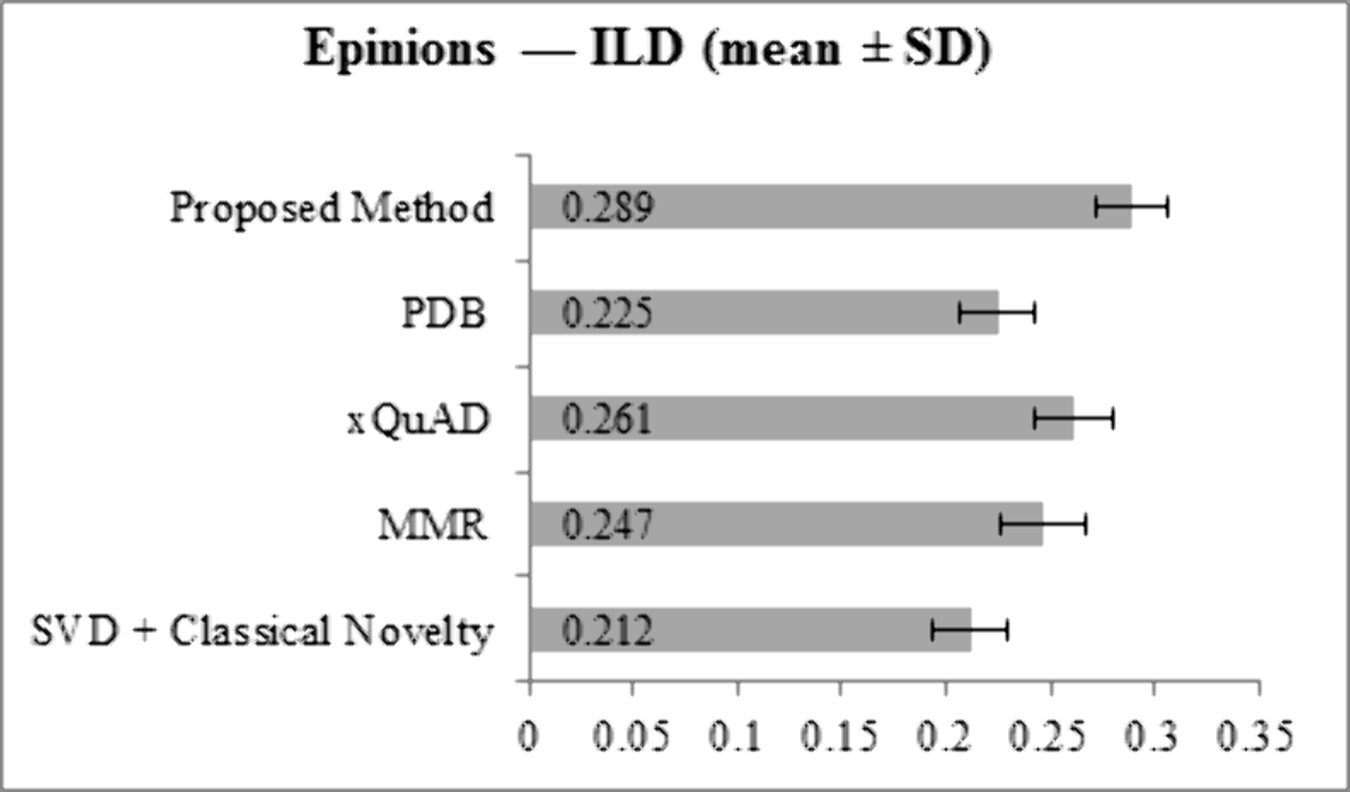
ILD results for Epinions dataset across all methods.

Figs 5 and 6 show ILD values for LastFM and Epinions. On LastFM, the proposed method achieves 0.315, outperforming all baselines: SVD + Classical Novelty (0.235), MMR (0.278), xQuAD (0.291), and PDB (0.250). On Epinions, the proposed method reaches 0.289, again exceeding SVD + Classical Novelty (0.212), MMR (0.247), xQuAD (0.261), and PDB (0.225). These results indicate that the proposed framework generates recommendation lists with higher item heterogeneity compared to all baseline methods.

**Fig 7:**
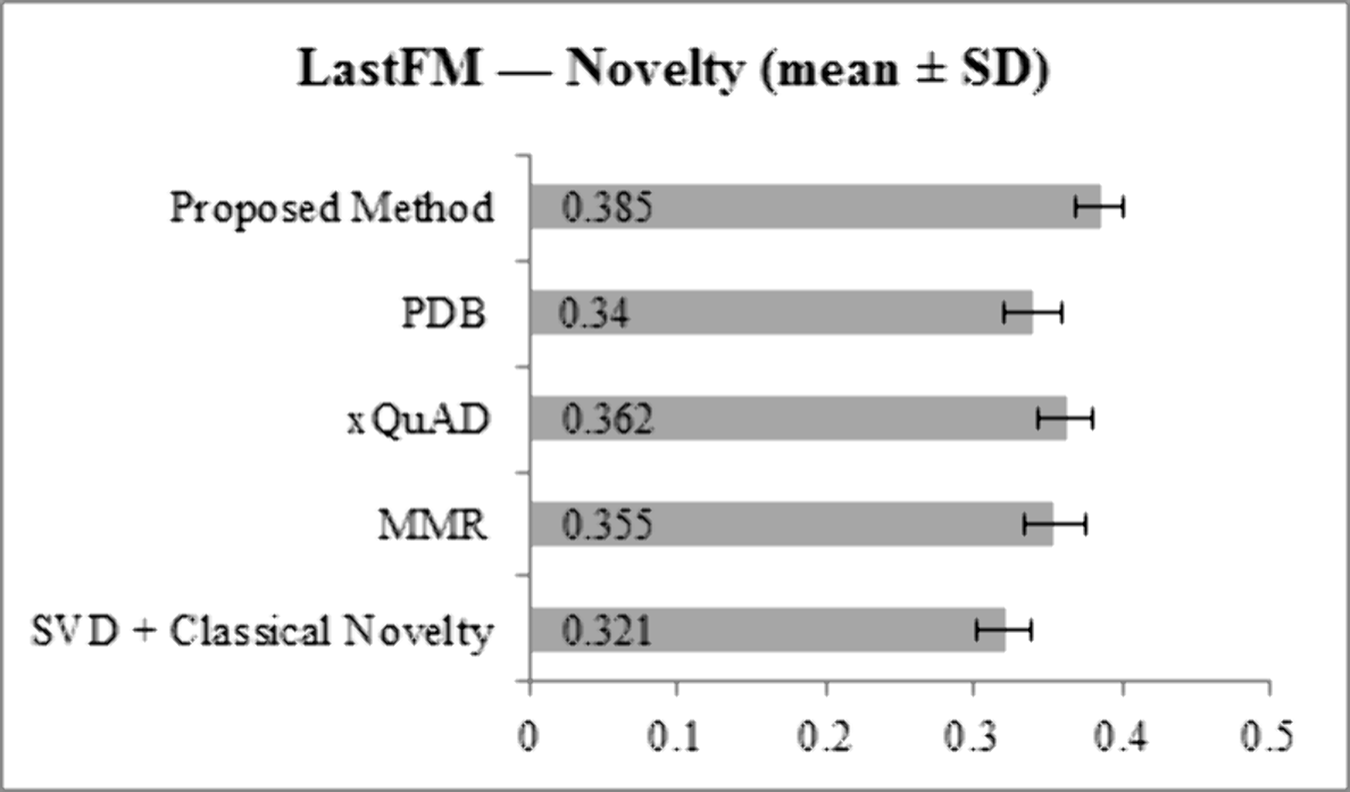
Novelty results for LastFM dataset across all methods.

**Fig 8:**
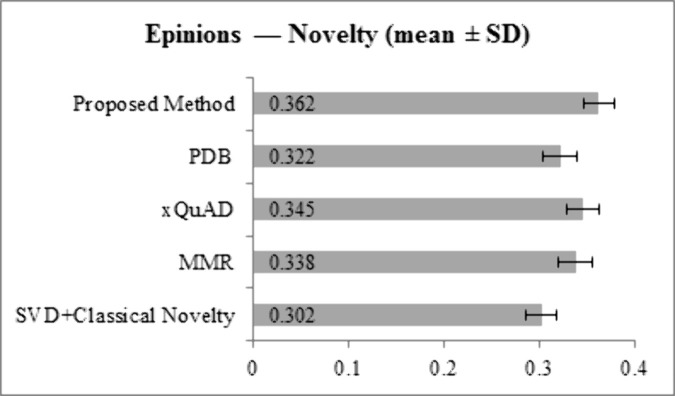
Novelty results for Epinions dataset across all methods.

Figs 7 and 8 illustrate Novelty scores. On LastFM, the proposed method achieves 0.385, higher than SVD + Classical Novelty (0.321), MMR (0.355), xQuAD (0.362), and PDB (0.340). On Epinions, the proposed method reaches 0.362, outperforming SVD + Classical Novelty (0.302), MMR (0.338), xQuAD (0.345), and PDB (0.322). This demonstrates the method’s ability to recommend less popular or previously unexplored items, aligning with the goal of personalized novelty.

**Fig 9:**
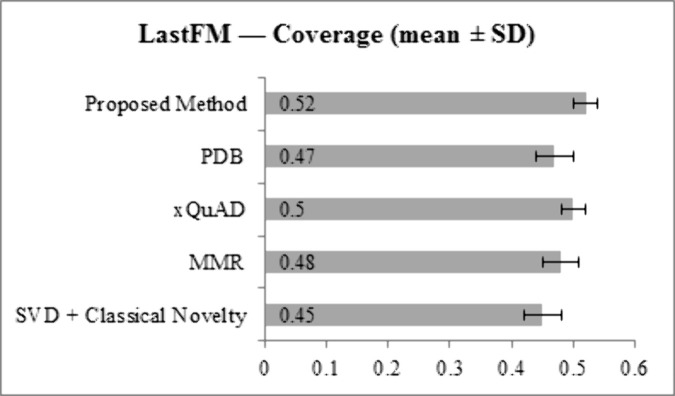
Coverage results for LastFM dataset across all methods.

**Fig 10:**
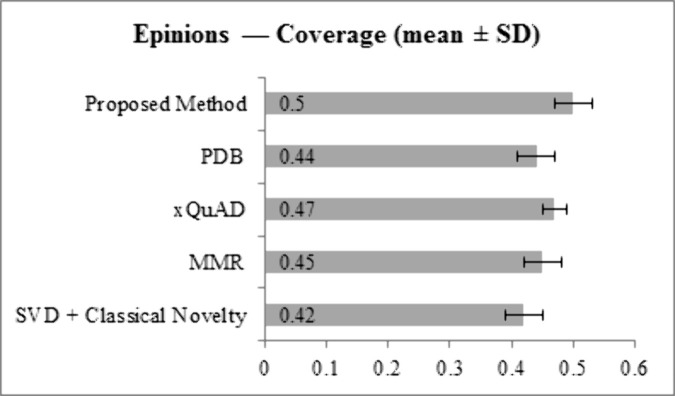
Coverage results for Epinions dataset across all methods.

Figs 9 and 10 present Coverage results. On LastFM, the proposed method achieves 0.52, higher than SVD + Classical Novelty (0.45), MMR (0.48), xQuAD (0.50), and PDB (0.47). On Epinions, Coverage reaches 0.50, exceeding SVD + Classical Novelty (0.42), MMR (0.45), xQuAD (0.47), and PDB (0.44). These results indicate broader utilization of the item catalog, with improved exposure of long-tail items compared to all baselines.

The figures collectively indicate that the proposed method outperforms all baseline approaches in ILD, Novelty, and Coverage, while maintaining competitive NDCG@10 values. These results highlight a balanced trade-off between ranking accuracy and exploration of novel content, confirming the effectiveness of the personalized novelty framework.

## 6. Discussion

The results demonstrate that the proposed personalized novelty framework effectively balances the trade-off between ranking accuracy and the exploration of diverse and novel items. Across both the LastFM and Epinions datasets, the proposed method consistently outperforms the four baseline approaches (SVD + Classical Novelty, MMR, xQuAD, and PDB) in Intra-List Diversity (ILD), Novelty, and Coverage, while maintaining competitive NDCG@10 scores. These findings indicate that incorporating social context and user-specific novelty preferences enables the model to generate recommendation lists that are both relevant and heterogeneous.

The slight reductions in NDCG@10 observed in some cases reflect the inherent trade-off between accuracy and increased diversity or novelty, a phenomenon widely reported in recommender system research. However, the observed decreases are minimal (0.01–0.02), suggesting that the proposed method preserves ranking quality while providing substantial improvements in metrics that enhance user discovery and engagement.

Compared to baseline re-ranking strategies, the proposed framework offers several advantages. Unlike classical popularity-based novelty approaches, it accounts for individual user behavior and social community structure, resulting in more personalized recommendations. Methods such as MMR and xQuAD focus on diversity and novelty from a global perspective and do not explicitly consider the interplay between user social connections and novelty preferences. Similarly, PDB addresses popularity bias but does not adapt novelty scoring to individual user profiles. In contrast, the proposed method dynamically integrates community-level item adoption patterns with personalized novelty scores, enabling more fine-grained and context-aware recommendations.

These improvements have direct practical implications for recommender systems. By effectively promoting long-tail items and increasing catalog coverage, the framework can enhance user engagement, reduce repetitive recommendations, and facilitate the discovery of less-explored content. These capabilities are particularly valuable in domains with extensive item catalogs or socially-influenced user behavior, such as music, e-commerce, and online review platforms.

Nevertheless, the study has some limitations. The method currently does not address cold-start users, as personalization relies on historical interactions within social communities. Additionally, the social graph is considered static within each evaluation period, which may not fully capture dynamic changes in user interactions over time. Future work could explore integration with temporal social network analysis, as well as combining the framework with content-based features or deep learning models to further enhance novelty personalization and recommendation accuracy.

In summary, the proposed personalized novelty framework demonstrates that user-centric and social context-aware modeling of novelty can significantly improve diversity, novelty, and coverage in recommender systems while maintaining competitive relevance. These results support the potential of the approach to enhance user satisfaction and content discovery in practical applications.

## 7. Conclusion and future work

In this study, we proposed a novel framework for personalizing novelty in recommender systems, leveraging both user behavior and social community structure to generate diverse and novel recommendations. Experimental results indicate that the proposed method:

Outperforms all four baseline approaches in ILD, Novelty, and Coverage,

Maintains competitive NDCG@10 scores with minimal reduction,

Enables personalized recommendations based on users’ social context and novelty preferences.

These findings suggest that integrating social context and individual novelty tendencies can enhance user experience, facilitate content discovery, and mitigate over-reliance on popular items in modern recommender systems. Future research could extend the framework to accommodate cold-start users, incorporate dynamic social graphs, and combine with content-based features or deep learning models to further improve both novelty personalization and recommendation accuracy. Another promising direction is the deployment of online recommender systems capable of adapting to real-time user feedback. Such systems can dynamically refine recommendations as user preferences evolve, leading to higher engagement and satisfaction. Moreover, future work can benefit from exploring Explainable Recommender Systems (XRS) to enhance transparency and user trust. By providing clear and user-friendly explanations for why specific items are recommended—especially in the context of novelty and personalization—users are more likely to understand and accept system suggestions. Additionally, integrating psychological user modeling approaches—such as personality profiling using frameworks like the Big Five Inventory (BFI), HEXACO, or MBTI—can provide a more nuanced understanding of user preferences. Personality-informed recommender systems have shown potential in tailoring content to users’ intrinsic traits, which can be particularly effective in improving the relevance and perceived novelty of recommendations. Combining these personality dimensions with interaction-based novelty modeling may yield richer and more adaptive recommendation strategies.

Finally, further investigation into the impact of social dynamics and contextual factors on user decision-making could uncover valuable insights, enabling the design of more socially aware and contextually adaptive recommender systems.
